# Transfer of *β*-hydroxy-*β*-methylbutyrate from sows to their offspring and its impact on muscle fiber type transformation and performance in pigs

**DOI:** 10.1186/s40104-016-0132-6

**Published:** 2017-01-07

**Authors:** Haifeng Wan, Jiatao Zhu, Caimei Wu, Pan Zhou, Yong Shen, Yan Lin, Shengyu Xu, Lianqiang Che, Bin Feng, Jian Li, Zhengfeng Fang, De Wu

**Affiliations:** Institute of Animal Nutrition, Sichuan Agricultural University, No. 211, Huimin Road, Wenjiang District, Chengdu, 611130 Sichuan People’s Republic of China

**Keywords:** *β-*hydroxy-*β*-methylbutyrate, Lactating sow, Muscle fiber, Offspring, Performance

## Abstract

**Background:**

Previous studies suggested that supplementation of lactating sows with *β*-hydroxy-*β*-methylbutyrate (HMB) could improve the performance of weaning pigs, but there were little information in the muscle fiber type transformation of the offspring and the subsequent performance in pigs from weaning through finishing in response to maternal HMB consumption. The purpose of this study was to determine the effect of supplementing lactating sows with HMB on skeletal muscle fiber type transformation and growth of the offspring during d 28 and 180 after birth. A total of 20 sows according to their body weight were divided into the control (CON, *n* = 10) or HMB groups (HMB, *n* = 10). Sows in the HMB group were supplemented with *β*-hydroxy-*β*-methylbutyrate calcium (HMB-Ca) 2 g /kg feed during d 1 to 27 of lactation. After weaning, 48 mixed sex piglets were blocked by sow treatment and fed standard diets for post-weaning, growing, finishing periods. Growth performance was recorded during d 28 to 180 after birth. Pigs were slaughtered on d 28 (*n* = 6/treatment) and 180 (*n* = 6/treatment) postnatal, and the longissimus dorsi (LD) was collected, respectively.

**Results:**

The HMB-fed sows during lactation showed increased HMB concentration (*P* < 0.05) in milk and LD of weaning piglets (*P* < 0.05). In addition, offsprings in HMB group had a higher finishing BW and lean percentage than did pigs in CON group (*P* < 0.05), meanwhile, compared with pigs from sows fed the CON diet, pigs from sows fed HMB diet showed higher type II muscle fiber cross-sectional area (CSA), elevated myosin heavy chain (MyHC) *IIb* and *Sox6* mRNA, and fast-MyHC protein levels in LD (*P* < 0.05).

**Conclusion*s*:**

HMB supplemented to sow diets throughout lactation increases the levels of HMB in maternal milk and skeletal muscle of pigs during d 28 after birth and promotes subsequent performance of pigs between d 28 and 180 of age by enhancing glycolytic muscle fiber transformation.

## Background

It has been suggested that establishing sufficient skeletal muscle is essential for lasting metabolic health [1]. Growing evidence has further indicated that maternal nutrition during lactation plays an important role in postnatal skeletal muscle development and growth of the offspring [2]. Skeletal muscle satellite cells provide myonuclei with muscle fibers and fuse with them, leading to muscle fiber hypertrophy. In addition, the transitions in myosin heavy chain isoforms are accelerated at postnatal 2 to 3 wk and muscle fiber type is quite vulnerable to various stimuli, which might affect subsequent growth and health [3]. Research has suggested that a high-protein/ low-carbohydrate diet fed to mice during lactation impeded skeletal muscle growth in offspring and caused a transient shift towards oxidative versus glycolytic muscle metabolism [4]. Meanwhile, Lefaucheur et al. [5] reported that early postnatal under-nutrition of piglets reduced the glycolytic capacity of skeletal muscle, which was reflected a delay in muscle maturation. In addition, studies have shown that insulin resistance correlates with skeletal muscle fiber distribution and a decreased percentage of slow oxidative type I fibers [6, 7]. Conde-Aguilera et al. also found that feeding a diet with 30% deficient in total sulfur amino acid to piglets during 10 d after weaning decreased fast-twitch muscle growth and glycolytic metabolism [8]. As a result, it is of great interest that maternal nutrition interventions during lactation are being developed to increase or maintain skeletal muscle mass, which might have a lasting impact on future performance.

Studies have suggested that leucine could activate the mammalian target of rapamycin (mTOR) to promote skeletal muscle protein synthesis in pigs [9]. Moreover, *β*-hydroxy-*β*-methylbutyrate (HMB) is a metabolite of leucine, which has also similar effects with leucine in promoting skeletal muscle growth [10]. Moore et al. [11] showed that when the early post-hatch poult was fed with 0.1% *β*-hydroxy-*β*-methylbutyrate calcium (HMB-Ca), HMB may improve muscle development via an increase in myogenic satellite cell mitotic activity. Meanwhile, previous studies have also shown that HMB could stimulate satellite cell proliferation and differentiation [12, 13], and dietary HMB supplementation to piglets during early postnatal period promoted skeletal muscle fiber development [14]. However, limited research existed evaluating the impact of maternal HMB consumption during lactation on skeletal muscle development of the offspring. Therefore, the study was to assess the effect of feeding HMB to sows during lactation on muscle fiber type transformation and performance of the offspring during d 28 and 180 after birth.

## Methods

All procedure including use and treatment of animals was in accordance with guidelines set by the Animal Care and Use Committee of Sichuan Agricultural University. The calcium salt (monohydrate) of *β*-hydroxy-*β*-methylbutyrate (purity 93%), was purchased from Jiangyin Sanyi Chemical Co., Ltd., (Jiangsu, China).

### Animal, diets and treatments

A total of 20 Landrace × Yorkshire (the third parity) pregnant sows with similar body weight (252.9 ± 3.6 kg) were used in the experiment. At d 110 of gestation, sows were moved to farrowing room and were also individually penned (2.06 m × 1.50 m), and all sows were randomly assigned to control (CON, *n* = 10) or experimental groups (HMB, *n* = 10) according to their body weights. All sows were fed 2.60 kg/d gestation diet from d 110 of gestation to parturition. After farrowing, the CON sows were fed a basal lactation diet (Table 1), and the sows in the HMB group were fed basal lactation diet supplemented with 2.0 g/kg of HMB-Ca. The feed level was progressively increased during the first 5 d of lactation, and was allowed free access to feed during the d 6 of lactation to weaning, and sow feed intake was record daily during lactation. In addition, each pig from each litter was individually weighted at birth. In addition, the litter size was standardized to 10 piglets per litter by cross-fostering within the treatment group beyond 24 h after birth, and piglets had free access to water and creep feed was not provided to piglets at any point during the lactation period. Male piglets were castrated on d 7 after birth and all piglets were weaned at 28 d of age.

**Table 1:** Composition and nutrient levels of the basal diets for sows during lactation (as-fed basis)^a^

Ingredient	Percent
Corn	63.50
Soybean meal	23.00
Fish meal	3.00
Wheat bran	5.00
Soybean oil	2.50
L-Lysine HCl (98%)	0.08
Limestone	0.87
Dicalcium phosphate	1.00
Choline chloride (50%)	0.15
Salt	0.40
Vitamin and mineral premix^b^	0.50
Calculated nutrient levels^c^	
CP, %^d^	17.90
ME, MJ /kg^d^	13.65
Ca, %^d^	0.78
TP, %^d^	0.63
AP, %^d^	0.41
Total Lys, %^e^	1.02
Total Met + Cys, %^d^	0.58
Total Leu, %^e^	1.51

^a^The HMB group diet was formed by supplementing 2.0 g/kg HMB-Ca to the basal diets

^b^Provided per kg of diet: copper, 20 mg; iron, 80 mg; zinc, 100 mg; manganese, 25 mg; selenium, 0.15 mg; iodine, 0.14 mg, vitamin A, 4000 IU; vitamin D_3_, 800 IU; vitamin E, 441 IU; menadione, 0.5 mg; thiamine, 1.0 mg; riboflavin, 3.75 mg; vitamin B_6_, 1.0 mg; vitamin B_12_, 15 μg; niacin, 10 mg; D-pantothenic acid, 12 mg; folic acid, 1.3 mg; D-biotin, 200 μg for gestation and lactation diet

^c^The nutrient composition of diets were calculated using nutrition value for the ingredients obtained from China Feed Information Database 2013 (24)

^d^Calculated values

^e^Analyzed values

At weaning (d 28 of lactation), due to housing restriction, a total of 48 mixed sex pigs (24 barrows and 24 gilts, *n* = 24/treatment) from ten randomly litters per treatment approaching an average weight were selected and blocked according to maternal treatment, and were moved to 12 nursery pen (1.46 m× 1.30 m per pen, 2 barrows and 2 gilts per pen) until d 66 of age. At the d 67 of age, these pigs were moved to 12 grow-finish pens (2.03 m× 1.90 m per pen, 2 barrows and 2 gilts per pen) until the d 180 of age. In the whole experimental period, all the pigs had free access to water and were fed ad libitum, and they were phased-fed standard nursery and growing-finishing diets twice daily until d 180 after birth (Table 2).

**Table 2:** Composition and nutrient levels of the basal diets for the offspring (as-fed basis)

	Growth stage			
	D 28 to 66	D 67 to 108	D 109 to 150	D 151 to 180
Ingredient, %				
Corn	59.6	64.3	70.4	75.3
Soybean meal	18.0	24.0	22.0	16.0
Fish meal	3.0	2.0	–	–
Full fat soybean	5.0	–	–	–
Wheat bran	–	5.0	4.0	5.0
Whey powder	8.0	–	–	–
Sucrose	2.0	–	–	–
L-Lysine HCl (98%)	–	0.26	0.28	0.27
D,L-Methionine (99%)	–	0.08	0.06	0.06
L-Threonine (98.5%)	–	0.06	0.06	0.06
Limestone	0.50	0.60	0.80	0.85
Dicalcium phosphate	1.00	1.30	1.00	1.06
Choline chloride (50%)	0.10	0.10	0.10	0.10
Salt	0.30	0.30	0.30	0.30
Soybean oil	1.00	1.00	–	–
Vitamin and mineral premix^a^	1.50	1.00	1.00	1.00
Calculated nutrient levels^b^				
ME, MJ/kg	13.39	13.35	13.19	13.15
CP, %	18.0	17.7	15.9	13.8
Ca, %	0.72	0.71	0.63	0.63
TP, %	0.60	0.67	0.55	0.54
Total Lys, %	1.35	1.12	0.97	0.84

^a^Provided per kg of diet for piglets from d 28 to 66 of age: iron, 100 mg; copper, 6 mg; zinc, 100 mg; manganese, 4 mg; selenium, 0.30 mg; and iodine, 0.14 mg; vitamin A, 2,﻿200 IU; vitamin D_3_, 220 IU; vitamin E, 16; menadione, 0.5 mg; thiamine, 1.5 mg; riboflavin, 4.0 mg; vitamin B_6_, 7.0 mg; vitamin B_12_, 20 μg; niacin, 30 mg; D-pantothenic acid, 12 mg; folic acid, 0.3 mg; and D-biotin, 80 μg Provided per kg of diet for pigs from d 67 to 180 of age: iron, 60 mg; copper, 6 mg; zinc, 60 mg; manganese, 4 mg; selenium, 0.30 mg; and iodine, 0.14 mg; vitamin A, 1,300 IU; vitamin D_3_, 150 IU; vitamin E, 11 IU; menadione, 0.5 mg; thiamine, 1.0 mg; riboflavin, 3.0 mg; vitamin B_6_, 1.0 mg; vitamin B_12_, 10 μg; niacin, 30 mg; D-pantothenic acid, 8 mg; folic acid, 0.3 mg; D-biotin, 50 μg

^b^The nutrient composition of diets were calculated using nutrition value for the ingredients obtained from China Feed Information Database 2013 (24)

### Performance measurement and sample collection

For the growth performance study, pigs were individually weighted at birth and postnatal d 28, 66, 108, 150 and 180, respectively. The average daily gain (ADG) was calculated as (weight change/number of days) ×1000. Average daily feed intake was obtained from all pens in the nursery period and growing–finishing period, and the Feed/Gain (F/G) was calculated as the average daily feed intake (ADFI) /ADG. Plasma samples (10 mL) were also collected from the jugular vein of six piglets closest to the average body weight of each treatment on d 28 of lactation. Samples were collected in heparinized tubes kept on the ice and centrifuged at 2,550 × g for 10 min at 4 °C. Milk samples (30 mL) were collected from all functional glands of sows at d 28 of lactation by injecting 1 mg oxytocin into the ear vein. The plasma supernatant and milk samples were refrigerated at −20 °C immediately prior to subsequent analysis. At d 28 and 180 after birth, a total of 24 pigs (one castrated male pig with mean body weight per pen) were slaughtered after overnight fasting by electrical stunning followed by exsanguination, according to the method of Refeldt et al. [4]. After slaughter, the abdomen was opened, and the entire intestine was rapidly removed. Then, all samples for mRNA, histological and biochemical analyses were collected as quickly as possible. Longissimus dorsi (LD) samples from the left half of the carcass were dissected at the level of the 12^th^/13^th^ ribs for pigs during d 28 and 180 after birth, as previously described by Cerisuelo et al. [15], and were rapidly frozen in liquid nitrogen and stored at −80 °C for subsequent RNA and biochemical analyses. The LD at the level of the 11^th^/12^th^ ribs was excised and stored in liquid nitrogen for subsequent muscle morphological assessment. The right half of the carcass was dissected into primary cuts, such as the loin, neck and ham, which were further manually separated into lean meat, subcutaneous fat, bones, and skin for pigs at d 180 after birth, as described by Rehfeldt et al. [4].

### Milk and muscle HMB determination

Milk and muscle tissue samples were measured for HMB content by a modification of the plasma and milk analysis method previously described by Deshpande et al. [16] and Ehling et al. [17]. Briefly, acidified muscle tissue homogenates were extracted with methyl-t-butyl ether for 2 h, and *a*-hydroxy-*a*-methylbutyric acid (Sigma H40009; Sigma-Aldrich, Saint Louis, MO, USA) was used as an internal standard. The extracting solution was centrifuged at 12,000 × *g* and 4 °C for 5 min. Then, the supernatant was transferred to a clean test tube and evaporated to dryness by nitrogen flushing at 40 °C. After drying, 1 and 4 mL of 0.10 mol/L HCl in 90/10 (v/v) water-acetonitrile were added the same test tube for milk and muscle sample, respectively. After a brief vortex mixing, the mixture was passed through an OASIS® MCX cartridge (60 mg) and the eluate was collected for the muscle sample, and this step was omitted for milk sample. The HMB content was analysed via high-performance liquid chromatography with tandem mass spectrometry (LC-MS/MS, Agilent, USA). The recovery intra-day and inter-day are all > 85 and 70% for sow milk and piglet skeletal muscle, respectively, and the detectable minimum amount of HMB in the sow milk and piglet skeletal muscle were 0.02 μmol/L or 0.02 nmol/g.

### Biochemical analyses and amino acid content analysis

Plasma insulin concentration was analyzed using a porcine insulin RIA kit (Tianjin Jiuding Medical Biological Engineering Co., LTD, Tianjin, China), and the detection limit of plasma insulin was 2 μU/mL. Plasma glucose and urea levels were conducted by using specific assay kits (Nanjing Jiancheng Bioengineering Institute, Nanjing, China) according to the manufacturer’s instructions, moreover, the detection range were 0–28 mmol/L and 0.03-19 mmol/L for plasma glucose and urea, respectively. Plasma free amino acids content were determined according to the method as described by Li et al. [18], and briefly, 300 μL of plasma sample and 900 μL of 10% sulfosalicylic acid were mixed well and centrifuged at 12, 000 × g and 4 °C for 15 min. Then the supernatant fluid was filtered through a 0.22-μm-pore-size PTFE syringe filter (Millpore) into a 2-mL auto-sampler vial and determined for amino acid level by an L8800 high-speed analyzer (Hitachi, Tokyo, Japan) for the amino acid. Amino acid standard solutions type B and AN-II (Wako Pure Chemical Industries, Ltd., Osaka, Japan) were used for calibration.

### Cross-sectional area and total number of muscle fiber determination

For histological analyses of muscle fiber, frozen muscle samples were equilibrated to −25 °C. Then transverse serial section (10 μm) was cut in a cryostat at −20 °C and subsequently mounted on glass microscopic slides. These sections were then stained with acid-preincubated ATPase treatment at pH 4.20 and subsequent alkaline (pH = 9.40) treatment to evaluate muscle fiber morphology using a modification of the method of Guth et al. [19]. All sections were photographed using a digital microscope (Nikon), and muscle fibers were counted over 5 randomly selected fields of known size (1.01 mm^2^, 200 to 300 fibers). The total number of fibers was calculated by multiplication of the number of fibers per centimeter^2^ with the loin meat area (LMA), and the mean muscle fiber cross-sectional area (CSA) in the united area was measured by Image-Pro Plus 6.0 software (Media Cybernetics, Bethesda, MD).

### RNA isolation, cDNA synthesis and real-time PCR

Approximately 50 mg of muscle samples were crushed and total RNA was extracted using RNAiso Plus reagent (TaKaRa). The integrity of RNA was estimated by electrophoresis on a 1% agarose gel stained with ethidium bromide and the purity of total RNA was verified using a Nanovue^TM^ Plus Spectrophotometer (GE Healthcare, UK) at 260 and 280 nm. The OD_260_/OD_280_ ratios of the RNA samples were all between 1.8 and 2.0. Subsequently, reverse transcription was performed from 1 μg of total RNA using the PrimeScript^TM^ RT Reagent Kit (TaKaRa) according to the manufacturer’s recommendations, and the reverse transcription products (cDNA) were stored at −20 °C for relative quantification by PCR. Primers were designed by Primer Express 3.0 (Applied Biosystems, Foster, CA, USA) based on known sequence deposited in GenBank, and the detected genes of muscle samples included myogenic genes (*Pax7*, *MRF4* and *MSTN*), protein synthesis and degradation factors (*IGF-I*, *mTOR, MAFbx* and *MuRF1*), transcription factor (*Sox6* and *FoxO1*), myosin heavy chain (*MyHC*) isoform (*slow/I, IIa, IIx and IIb*), and the information of primers was presented in Table 3. Real-time-qPCR analysis was carried out using the SYBR Green method and the target genes were quantified using CFX manager V1.1 software (Bio-Rad Laboratories). The mixture (10 μL) contained 5 μL SYBR® Premix Ex Taq^TM^ II (TaKaRa), 1 μL cDNA, 0.5 μL of each gene-specific primer, and 3 μL ddH_2_O. All measurements were performed in triplicate. The thermal cycling conditions were follows: denaturation at 95 °C for 15 s, followed by 40 cycles of denaturation at 95 °C for 5 s and annealing at 61.5 °C for 30 s, and collection of fluorescent signals. The relative mRNA abundances of the target genes were calculated using the 2^-ΔΔCT^ method, and glyceraldehyde-3-phosphate dehydrogenase (*GAPDH*) was used as a reference gene in this study. The mRNA level of each target gene for Con group was set to 1.0.

**Table 3:** The primer sequences of the target genes

Target gene	Primer sequence (5′to 3′)	Product size, bp	GenBank No.
*MyHC1*	F: GTTTGCCAACTATGCTGGGG	95	NM_213855.1
	R: TGTGCAGAGCTGACACAGTC		
*MyHCIIa*	F: CTCTGAGTTCAGCAGCCATGA	127	NM_214136.1
	R: GATGTCTTGGCATCAAAGGGC		
*MyHCIIx*	F: TTGACTGGGCTGCCATCAAT	111	NM_001104951.1
	R: GCCTCAATGCGCTCCTTTTC		
*MyHCIIb*	F: GAGGTACATCTAGTGCCCT	83	NM_001123141.1
	R: GCAGCCTCCCCAAAAATAGC		
*Pax7*	F: TGAAGGTCGGAGTGAACGGAT	74	NM_001206359.1
	R: CACTTTGCCAGAGTTAAAAGCA		
*IGF-1*	F: CACAGACGGGCATCGTGGAT	90	NM_214256.1
	R: ACTTGGCAGGCTTGAGGGGT		
*mTOR*	F: CATTGGAGATGGTTTGGTGA	160	XM_003127584.5
	R: ATGGGATGTGGCTTGTTTGA		
*MRF4*	F: CCTTCGGTGCCTTTCTTCCAT	88	NM_001244672.1
	R: GAGTTATTTCTCCCCCACTTCC		
*MSTN*	F: TTTACCTGTTTATGCTGATTGTTG	194	NM_214435.2
	R: TTTGCTAATGTTAGGAGCTGTTTC		
*FoxO1*	F: TCAAGGATAAGGGCGACAGC	95	NM_214014.2
	R: AATGTCATTATGGGGAGGAGAGT		
*Sox6*	F: CGGATTGGGGAGTATAAGCA	159	XM_01399454.1
	R: CATCTGAGGTGATGGTGTGG		
*MAFbx*	F: CCCTCTCATTCTGTCACCTTG	104	NM_001044588
	R: ATGTGCTCTCCCACCATAGC		
*MuRF1*	F: GCTGGATTGGAAGAAGATGTAT	144	NW_001184756
	R: AGGAAAGAATGTGGCAGTGTCT		
*GAPDH*	F: TGAAGGTCGGAGTGAACGGAT	74	NM_001206359.1
	R: CACTTTGCCAGAGTTAAAAGCA		

*MyHC* myosin heavy chain, *Pax7* paired box 7, *IGF-I* insulin-like growth factor-I, *mTOR* mammalian target of rapamycin, *MRF4* muscle regulator factor 4, *MSTN* myostatin, *FoxO1* forkhead box transcription factor O1, *MAFbx* muscle atrophy F-box, *MuRF1* muscle Ring finger 1, GAPDH glyceraldehyde-3-phosphate dehydrogenase

### Tissue protein extraction and Western blot analysis

Frozen LD samples were lysed, and the protein concentration was measured using a protein assay kit (Nanjing Jiancheng Bioengineering Institute, Nanjing, China) according to the manufacturer’s instructions. Western blot analysis of fast-MyHC protein (anti-fast myosin skeletal heavy chain antibody, Abcam, ab91506, diluted 1: 5,000) was conducted according to a previous publication [20]. The relative expression of the target protein was normalized to GAPDH (bio-rich042, BMSX, diluted 1: 20,000) as an internal control. The band density of fast-MyHC was normalized to that of GAPDH and fast-MyHC protein content was presented as the fold change relative to the CON group.

### Statistical analysis

For performance of fattening pigs, individual pen served as the experimental unit, and other data including skeletal muscle characteristics, plasma amino acid and metabolites, milk and muscle HMB levels, muscle fiber histological analyses, gene and protein expressions were analyzed with the individual pig as the experimental unit. For means separation, differences among the least square means of the CON vs. HMB treatments were compared using *t*-tests analysis. The results for mRNA level and fast-MyHC protein expression are presented as the least square mean and their SEM, and other results in tables are shown as the mean and pooled SEM. A difference was considered statistically significant when *P* < 0.05, if the *P*-values were between 0.05 and 0.10, the difference was considered a tendency.

## Results

### Performance and skeletal muscle composition

**Fig. 1:**
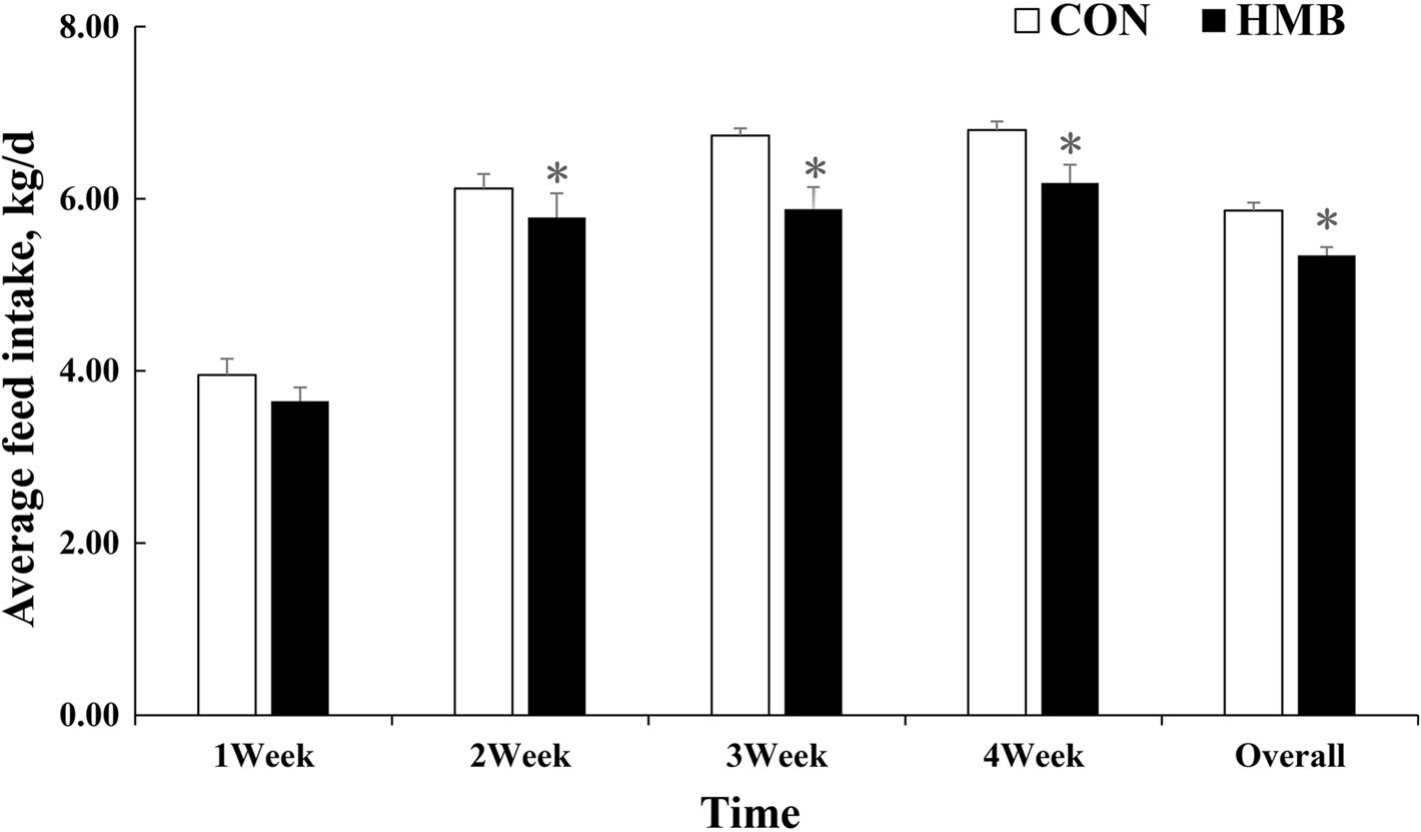
Effects of dietary supplementation of HMB on average feed intake of sows during lactation. Values are means, with their standard errors represented by vertical bars (*n* = 10). *Represents mean values between the two groups differ significantly at *P* < 0.05. CON control; HMB *β*-hydroxy-*β*-methylbutyrate

The results of sow feed intake during lactation was provided in Fig. 1. Sows in the HMB group had lower feed intake than those in the CON group (*P* < 0.05). The fattening pig performance body composition are presented in Tables 4 and 5. The average feed intake (ADFI) of pigs during d 28 to 180 was not different between treatments. However, pigs from sows fed HMB diet during lactation exhibited higher final weight (+6.0%, *P* < 0.05), ADG (+6.4%, *P* < 0.05), and had a decreased tendency in F/G (−3.5%, *P* = 0.069) than did pigs from sows fed the CON diet during postnatal d 28 to 180. In addition, fattening pigs from sows fed HMB diets also showed superior carcass weight (*P* < 0.05) and lean meat percentage (*P* = 0.07) than did pigs from sows fed the CON diet.

**Table 4:** Effects of feeding HMB to sows during lactation on performance of the offspring

	CON	HMB	SEM	*P* value
Number of pigs on trial (n)	24	24		
Pig BW, kg				
At birth	1.41	1.42	0.07	0.887
D 28	7.65	7.81	0.32	0.670
D 66	22.6	22.9	0.5	0.677
D 108	47.6	49.4	1.1	0.293
D 150	79.8	83.9	1.4	0.080
D 180	105.7	112.0	1.8	0.041
Pig ADFI, g/d				
D 28 to 66	586	579	24	0.836
D 67 to 108	1,087	1,158	29	0.122
D 109 to 150	2,187	2,250	64	0.505
D 151 to 180	3,048	3,071	83	0.845
Overall	1,652	1,693	30	0.368
Pig ADG, g/d				
D 28 to 66	394	398	13	0.828
D 67 to 108	588	631	19	0.153
D 109 to 150	773	820	27	0.247
D 151 to 180	864	939	40	0.226
Overall	645	686	11	0.031
F/G				
D 28 to 66	1.49	1.45	0.03	0.457
D 67 to 108	1.85	1.84	0.03	0.720
D 109 to 150	2.83	2.74	0.05	0.200
D 151 to 180	3.56	3.28	0.15	0.232
Overall	2.56	2.47	0.03	0.069

*ADG* average daily gain, *BW* body weight, *ADFI* average daily feed intake, *CON* control, *HMB β*-hydroxy-*β*-methylbutyrate

**Table 5:** Effects of feeding HMB to sows during lactation on skeletal muscle composition of the offspring

	CON	HMB	SEM^a^	*P* value
Weaning pigs				
Body weight, kg	7.83	7.98	0.27	0.705
LD weight, g	127.2	130.7	9.0	0.792
Finishing pigs				
Body weight, kg	106.8	114.0	1.2	0.002
Carcass weight, kg	77.8	83.8	0.9	0.001
LD weight, kg	3.02	3.27	0.09	0.065
Lean meat, %	63.05	66.25	1.09	0.067
Bone, %	10.67	10.66	0.45	0.983
SCAT, %	20.01	16.21	1.19	0.051
Skin, %	6.27	6.88	0.23	0.100

*LD* longissimus dorsi, *SCAT* subcutaneous adipose tissue, *CON* control, *HMB β*-hydroxy-*β*-methylbutyrate

^a^Pooled standard error of the mean (*n* = 6/treatment)

### Milk and muscle HMB, plasma amino acid and metabolites concentrations

As shown in Table 6, sows supplemented with HMB diet had an increased HMB concentration in milk at d 28 of lactation (*P* < 0.01) than sows fed with CON diet, with piglets from HMB-supplemented sows having an elevated HMB concentration (*P <* 0.01) of LD than those of their CON counterparts at d 28 of age. In addition, in comparison with CON piglets, maternal HMB treatment increased plasma leucine (*P* < 0.05) and essential amino acid (EAA) contents (*P <* 0.05) of the piglets at d 28 of age. Similarly, plasma glucose content exhibited an increased level (*P* < 0.05) for piglets from HMB than CON groups, Furthermore, there was an increased tendency in the plasma insulin content for piglets from sows supplemented HMB diet than those from sows supplemented CON diet (*P* = 0.078).

**Table 6:** Effects of feeding HMB to lactating sows on HMB and amino acid metabolism in piglets during d 28 of lactation

	CON	HMB	SEM^a^	*P* value
HMB level				
Maternal milk, μmol/L	0.42	10.50	0.01	<0.001
Piglet’ LD, nmol/g tissue	6.04	7.51	0.33	0.025
Plasma metabolite				
Insulin, μU/mL	20.98	23.98	1.05	0.078
Glucose, mmol/L	7.71	9.77	0.46	0.029
Urea, mmol/L	4.79	4.40	0.34	0.860
Leucine, nmol/mL	155.6	227.7	20.8	0.044
BCAA, nmol/mL	516.8	561.2	37.8	0.437
EAA, nmol/mL	1,449	2,521	92	0.002
NEAA, nmol/mL	2,389	2,690	182	0.319

*LD* longissimus dorsi, *BCAA* branched-chain amino acids, *EAA* essential amino acid, *NEAA* nonessential amino acids, *CON* control, *HMB β*-hydroxy-*β*-methylbutyrate

^a^Pooled standard error of the mean (*n* = 10/treatment for maternal milk; *n* = 6/treatment for piglet’s LD and plasma metabolite)

### Muscle fiber characteristics

Data on the measures of CSA and numbers of muscle fiber in LD of fattening pigs are shown in Table 7. An increased tendency in the loin meat area (LMA) of LD was observed in fattening pigs from sows fed HMB diet compared with fattening pigs from sows fed the CON diet (*P* = 0.069). Similarly, higher type II muscle fiber CSA (*P* < 0.05) was discovered in offspring from sows fed HMB diet during lactation than in those from sows fed the CON diet, and pigs the HMB group had also an elevated tendency in the total mean myofiber CSA than those in the CON group (*P* = 0.065).

**Table 7:** Effects of feeding HMB to lactating sows on muscle fiber characteristics of fattening pigs

	CON	HMB	SEM^a^	*P value*
LMA, cm^2^	75.30	82.15	2.38	0.069
Total number of muscle fiber, ×10^3^	1013	1018	42	0.935
Mean myofiber CSA, μm^2^				
Total	3,615	4,268	183	0.065
Type I	2,732	3,203	213	0.169
Type II	3,719	4,774	252	0.025

*LMA* loin meat area, *CSA* cross sectional area, *CON* control, *HMB β*-hydroxy-*β*-methylbutyrate

^a^Pooled standard error of the mean (*n* = 6/treatment)

### Gene and protein expression

**Fig. 2:**
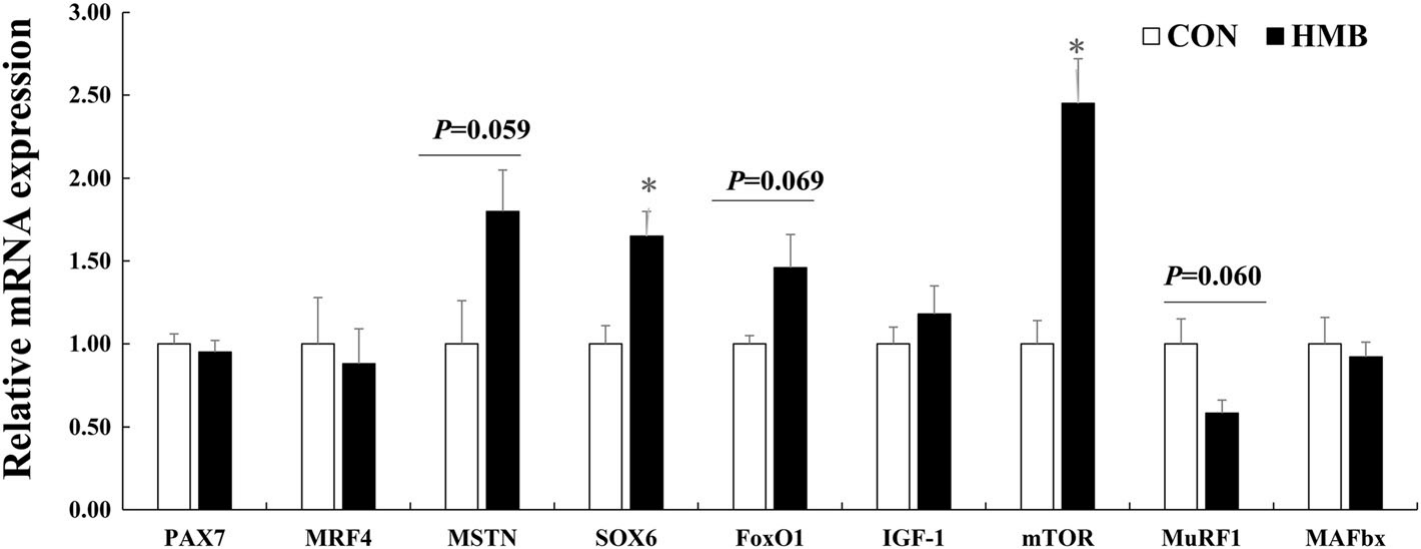
Effects of feeding HMB to lactating sows on myogenic, protein synthetic and proteolytic gene expressions in skeletal muscle of weaning piglets. Values are means, with their standard errors represented by vertical bars (*n* = 6). * Represents mean values between the two groups differ significantly at *P* < 0.05. Data was normalized against *GAPDH*, with results expressed relative to the CON sample using the ΔΔCt method (where Ct is cycle threshold) with efficiency correction. *Pax7* paired box 7; *IGF-I* insulin-like growth factor-I; *mTOR* mammalian target of rapamycin; *MRF4* muscle regulator factor 4; *MSTN* myostatin; *FoxO1* forkhead box transcription factor O1; *MAFbx,* muscle atrophy F-box; *MuRF1,* muscle Ring finger 1; CON control; HMB *β*-hydroxy-*β*-methylbutyrate

**Fig. 3:**
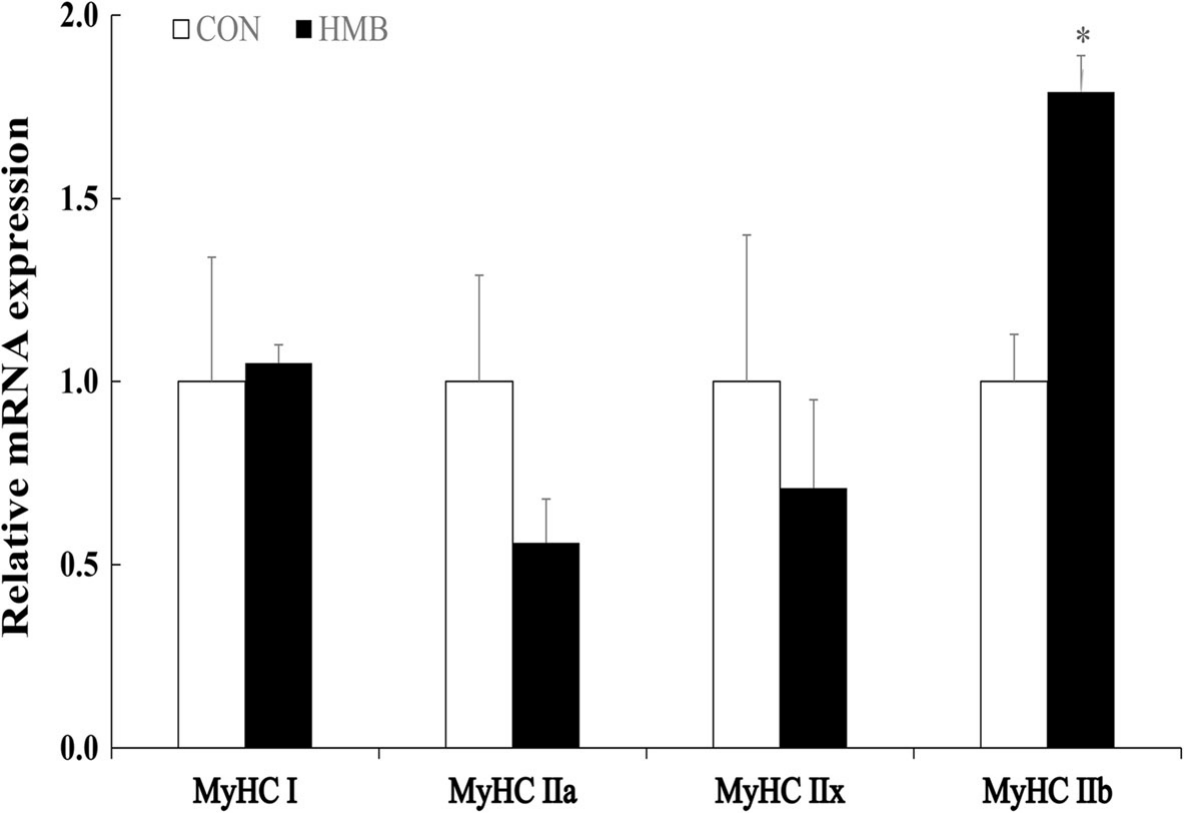
Effects of supplementing lactating sows with HMB on MyHC isoforms mRNA levels in skeletal muscle of the offspring at d 28 of age. Values are means, with their standard errors represented by vertical bars (*n* = 6). * Represents mean values between the two groups differ significantly at *P* < 0.05. Data was normalized against *GAPDH*, with results expressed relative to the CON sample using the ΔΔCt method (where Ct is cycle threshold) with efficiency correction. *MyHC* myosin heavy chain; CON control; HMB *β*-hydroxy-*β*-methylbutyrate

**Fig. 4:**
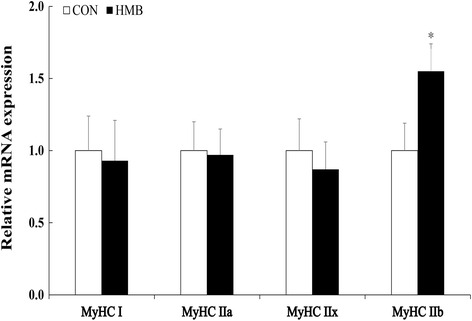
Effects of feeding HMB to lactating sows on MyHC isoforms mRNA level in skeletal muscle of the offspring at d 180 of age. Values are means, with their standard errors represented by vertical bars (*n* = 6). * Represents mean values between the two groups differ significantly at *P* < 0.05. Data was normalized against *GAPDH*, with results expressed relative to the control sample using the ΔΔCt method (where Ct is cycle threshold) with efficiency correction. *MyHC* myosin heavy chain; CON control; HMB *β*-hydroxy-*β*-methylbutyrate

**Fig. 5:**
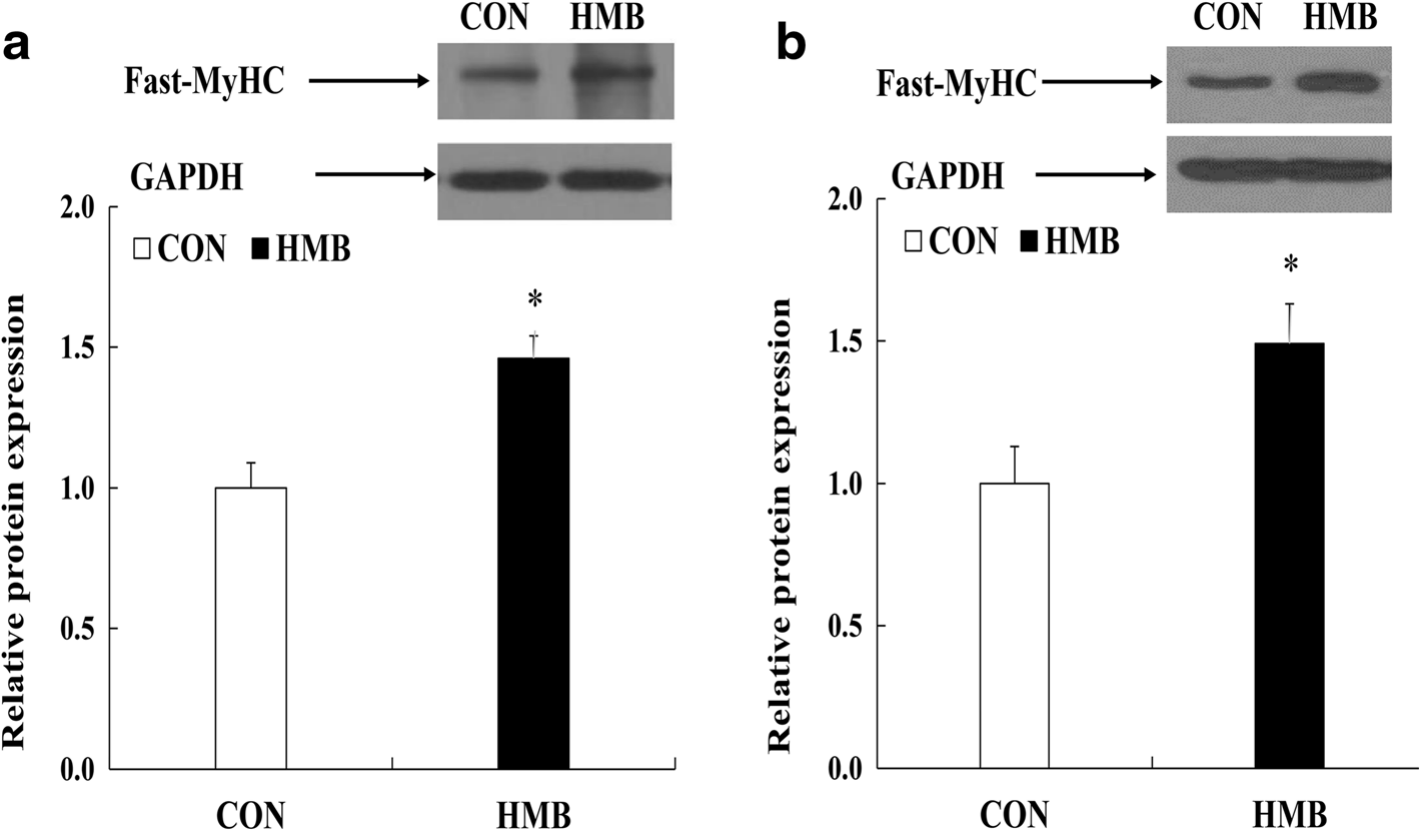
Effects of feeding HMB to lactating sows on fast-MyHC protein expression in skeletal muscle of the offspring during d 28 and 180 after birth. Values are means, with their standard errors represented by vertical bars (*n* = 6). *Represents mean values between the two groups differ significantly at *P* < 0.05. **a** (the offspring during d 28 after birth); **b** (the offspring during d 180 after birth); MyHC myosin heavy chain; GAPDH glyceraldehyde-3-phosphate dehydrogenase. CON control; HMB *β*-hydroxy-*β*-methylbutyrate

According to Figs. 2, 3, 4 and 5, maternal HMB treatment during lactation induced increased *mTOR* and *Sox6* mRNA levels in LD of the offspring at d 28 of age (*P* < 0.05). Meanwhile, no significant effects were observed in of mRNA levels of *MAFbx* and *MuRF1* in skeletal muscle of weaning piglets between treatments (*P* > 0.05). However, higher mRNA levels of *MyHC-IIb* were observed in LD of offspring in HMB group than those in the CON group during weaning and finishing stages (*P <* 0.05). In addition, weaning pigs in HMB group had an increased tendency in mRNA rate of *MyHC-IIb* /*MyHC-I* of LD than those in the CON group (*P* = 0.073). Similarly, compared with those in pigs in the CON group, pigs in HMB group exhibited higher the fast-MyHC protein levels in LD of the pigs at weaning and finishing stages (*P* < 0.05).

## Discussion

The first aim of the present study was to investigate the effects of maternal HMB consumption during lactation on performance and skeletal muscle characteristics of the offspring at weaning (d 28 after birth). It has established that maternal nutrition during lactation has previously been reported as one of the important factors affecting the growth of weaning pigs [21]. Given that pig performance was, to a great extent, influenced by birth BW, therefore, piglets with similar birth BW were selected and used in this experiment for CON and HMB groups, respectively. However, in the current trial, we found that maternal HMB treatment did not affect the BW of pigs at weaning, which disagreed with the findings of Nissen et al. [22], who reported that piglets from sows fed total HMB-Ca level of 2.0 g/d from d 108 of gestation to d 21 of lactation had higher weaning BW compared with those from sows fed the CON diet, and the discrepancy are likely related to the higher dosage (10.6 g/d) by multiplying dietary HMB level (2 g/kg) by average feed intake of sows (5.3 kg /d) during d 1 to 27 of lactation in this study compared with the used dosage (2 g/d) in previous study by Nissen et al. [22]. In addition, we found that dietary supplementation of higher level of HMB significantly reduced feed intake of sows during lactation (Fig. 1), which might be the important proof leading to the non-difference in BW of piglets at weaning. But till now it has not been demonstrated in the reduced feed intake of sows fed HMB, and research in the rat has shown that dietary HMB supplementation could impair insulin resistance and increase plasma non-essential fatty acid level [23], which could lead to the decreased feed intake during lactation, and the underlying mechanism of which remains to be explained further. However, studies have suggested that neonatal pigs have great growth potential in the immediate neonatal period, especially in terms of skeletal muscle growth, which is dependent on maternal milk intake and quality [24] and the increased milk production is associated with an increased lactation feeding level [25, 26], therefore, lower feed intake of sows during lactation might affect to some extent piglet growth in this study.

However, we found that feeding HMB to sows during lactation increased HMB level in maternal milk and LD of piglets in this study, which is consistent with the result of Nissen et al. [22] and Michelle et al. [27]. Meanwhile, higher plasma leucine, glucose, and insulin levels were observed in offspring in the HMB group, which is accordance with the results of Tatara et al. [28] and Wan et al. [14], and HMB could increase plasma amino acid and glucose levels of pigs [14, 28]. In this respect, insulin, glucose and amino acid were the important anabolic promoters affecting skeletal muscle protein synthesis [24], moreover, no difference were observed in mRNA expressions of *MuRF1* and *MAFbx*, which also provided the further evidence of the similar protein degradation in skeletal muscle of pigs between treatments. On the contrary, the increased mTOR mRNA expression was observed in the skeletal muscle of piglets in the HMB group. Therefore, these results suggested that HMB could not lead to the protein degradation of skeletal muscle in piglets in this study. In addition, It is appeared that the rate of *MyHC-II* mRNA level is positive to skeletal muscle maturity of piglets [5], in this experiment, maternal HMB treatment significantly increased *MyHC*-*IIb* mRNA level and fast-MyHC protein expression in LD of the offspring at weaning. Therefore, supplementation of sows with HMB during lactation might promote skeletal muscle transformation by transfer of HMB from maternal milk to skeletal muscle of the offspring during early postnatal period.

The secondary aim of this study was to explore the subsequent performance and skeletal muscle growth of pigs during finishing stage in response to maternal HMB consumption during lactation. Interestingly, an important observation was that pigs from sows in HMB group had greatly higher finishing BW compared with pigs from sows in the CON group, which was similar with the results of Tatara et al. [28, 29], who showed that when sow supplemented daily with 0.05 g/kg BW of HMB-Ca during the last two weeks of gestation, fattening pigs exhibited higher final BW at d 180 of age. Meanwhile, we found no statistical difference in ADFI between treatments from the weaning to d 180 after birth. Moreover, we also found that maternal HMB treatment contributed to an elevated carcass weight and lean meat percentage of pigs at d 180 of age. In this respect, a previous study found that feeding HMB to sows during lactation improved the performance of weaning pigs and increased HMB concentration in milk during d 20 of lactation, which are responsible for the enhanced skeletal muscle development of the offspring [22]. In addition, some studies also showed that pigs with heavier body weights exhibited greater muscle fiber cross-sectional areas and /or a greater number of muscle fibers than did pigs with lighter body weights at the same age [30]. A higher mean cross-sectional area of type II fiber was observed in LD of the offspring from sows fed HMB diet during lactation in our study, which also provided the important evidence for the improvement of performance by changing muscle fiber size of pigs. Therefore, it is possible that the increase in muscle fiber size in LD of pigs directly contributes to skeletal muscle growth and performance at d 180 after birth.

In addition, research has suggested that *MyHC-IIb* is the determining fiber contributing to the differentiation of large and small muscle area in the pig, and high muscularity is positively correlated with a high abundance of *MyHC-IIb* transcript [31], and more oxidative fibers might convert to glycolytic fibers with increasing age or weight, and that the early developmental stage might be a key stage for this conversion [32]. In this experiment, we found that maternal HMB treatment during lactation greatly increased *MyHC-IIb* mRNA and fast-MyHC protein levels in LD of the offspring at weaning and finishing stages (Figs. 3, 4, 5), which led to the improved subsequent performance of fattening pigs. Similarly, Rehfeldt et al. [33] reported that high live weight pig had more proportion of fast-twitch glycolytic fiber in semitendinosus muscle compared with low live weight pig by maternal daidzein feeding. Moreover, in this study, higher level of *Sox6* mRNA expression was observed in LD of weaning pigs from sows fed HMB diet during lactation. At present, there are few related studies in this respect. But, Wen et al. and Quiat et al. reported that overexpression of *Sox6* down-regulated *MyHC-I* expression and up-regulated *MyHC-IIb* expression in pig and mice [34, 35]. Based on the above results and the previous reports, it is possible that the increased HMB level of skeletal muscle accelerated *MyHC-IIb* isoform transformation of piglets during lactation, which might contribute to skeletal muscle growth during post-weaning stages. Further studies should be conducted in relation to the specific mechanisms of fast-twitch fiber development that are mediated by HMB in piglets.

## Conclusion

In conclusion, the present study showed that HMB supplemented to sows during lactation increases the levels of HMB in maternal milk and skeletal muscle of the weaning pigs, which contributes to the *MyHC-IIb* isoform transformation in muscle fiber of piglets at weaning, and subsequent performance of the offspring between d 28 and 180 of age.

## Abbreviations

CSA: Cross-sectional area
FoxO1: Forkhead box transcription factor O1
GAPDH: Glyceraldehyde-3-phosphate dehydrogenase
HMB: *β*-hydroxy-*β*-methylbutyrate
HMB-Ca: *β*-hydroxy-*β*-methylbutyrate calcium
IGF-I: Insulin-like growth factor-I
LC-MS/MS: Liquid chromatography with tandem mass spectrometry
LD: Longissimus dorsi
LMA: Loin meat area
*MAFbx*: Muscle atrophy F-box
MRF4: Muscle regulator factor 4
MSTN: Myostatin
mTOR: Mammalian target of rapamycin
*MuRF1*: Muscle Ring finger 1
MyHC: Myosin heavy chain
Pax7: Paired box 7

## References

[1] Brown LD (2014). Endocrine regulation of fetal skeletal muscle growth: impact on future metabolic health. J Endocrinol.

[2] Bayol SA, Bruce CR, Wadley GD (2014). Growing healthy muscles to optimise metabolic health into adult life. J Dev Org Health Dis.

[3] Lefaucheur L, Gerrard D (2000). Muscle fiber plasticity in farm mammals. J Anim Sci.

[4] Rehfeldt C, Stabenow B, Pfuhl R, Block J, Nurnberg G, Otten W (2012). Effects of limited and excess protein intakes of pregnant gilts on carcass quality and cellular properties of skeletal muscle and subcutaneous adipose tissue in fattening pigs. J Anim Sci.

[5] Lefaucheur L, Ecolan P, Barzic YM, Marion J, Le Dividich J (2003). Early postnatal food intake alters myofiber maturation in pig skeletal muscle. J Nutr.

[6] Oberbach A, Bossenz Y, Lehmann S, Niebauer J, Adams V, Paschke R (2006). Altered fiber distribution and fiber-specific glycolytic and oxidative enzyme activity in skeletal muscle of patients with type 2 diabetes. Diabetes Care.

[7] Gaster M, Staehr P, Beck-Nielsen H, Schrøder HD, Handberg A (2001). GLUT4 is reduced in slow muscle fibers of type 2 diabetic patients is insulin resistance in type 2 diabetes a slow, type 1 fiber disease?. Diabetes.

[8] Conde-Aguilera JA, Lefaucheur L, Tesseraud S, Mercier Y, Le Floc’h N, van Milgen J. Skeletal muscles respond differently when piglets are offered a diet 30% deficient in total sulfur amino acid for 10 days. Eur J Nutr. 2015;1–10. 10.1007/s00394-014-0830-9 25573689

[9] Boutry C, El-Kadi SW, Suryawan A, Wheatley SM, Orellana RA, Kimball SR (2013). Leucine pulses enhance skeletal muscle protein synthesis during continuous feeding in neonatal pigs. Am J Physiol Endocrinol Metab.

[10] Wilkinson DJ, Hossain T, Hill DS, Phillips BE, Crossland H, Williams J (2013). Effects of leucine and its metabolite beta-hydroxy-beta-methylbutyrate on human skeletal muscle protein metabolism. J Physiol.

[11] Moore DT, Ferket PR, Mozdziak PE (2005). The effect of early nutrition on satellite cell dynamics in the young turkey. Poult Sci.

[12] Kornasio R, Riederer I, Butler-Browne G, Mouly V, Uni Z, Halevy O (2009). Beta-hydroxy-beta-methylbutyrate (HMB) stimulates myogenic cell proliferation, differentiation and survival via the MAPK/ERK and PI3K/Akt pathways. BBA-Mol Cell Res.

[13] Alway SE, Pereira SL, Edens NK, Hao Y, Bennett BT (2013). Beta-Hydroxy-beta-methylbutyrate (HMB) enhances the proliferation of satellite cells in fast muscles of aged rats during recovery from disuse atrophy. Exp Gerontol.

[14] Wan H, Zhu J, Su G, Liu Y, Hua L, Hu L, et al. Dietary supplementation with *β*-hydroxy-*β*-methylbutyrate calcium during the early postnatal period accelerates skeletal muscle fibre growth and maturity in intra-uterine growth-retarded and normal-birth-weight piglets. Br J Nutr. 2016;1–10. 10.1017/S0007114516000465 26917333

[15] Cerisuelo A, Baucells MD, Gasa J, Coma J, Carrion D, Chapinal N (2009). Increased sow nutrition during midgestation affects muscle fiber development and meat quality, with no consequences on growth performance. J Anim Sci.

[16] Deshpande P, Jie Z, Subbarayan R, Mamidi VK, Chunduri RH, Das T (2013). Development and validation of LC-MS/MS method for the estimation of beta-hydroxy-beta-methylbutyrate in rat plasma and its application to pharmacokinetic studies. Biomed Chromatogr.

[17] Ehling S, Reddy TM (2014). Investigation of the presence of *β*-hydroxy-*β*-methylbutyric Acid and α-hydroxyisocaproic acid in bovine whole milk and fermented dairy products by a validated liquid chromatography–mass spectrometry method. J Agric Food Chem.

[18] Li H, Wan H, Mercier Y, Zhang X, Wu C, Wu X (2014). Changes in plasma amino acid profiles, growth performance and intestinal antioxidant capacity of piglets following increased consumption of methionine as its hydroxy analogue. Br J Nutr.

[19] Guth L, Samaha FJ (1970). Procedure for the histochemical demonstration of actomyosin ATPase. Exp Neurol.

[20] Wang J, Li X, Yang X, Sun Q, Huang R, Xing J (2011). Maternal dietary protein induces opposite myofiber type transition in Meishan pigs at weaning and finishing stages. Meat Sci.

[21] Ramanau A, Kluge H, Spilke J, Eder K (2004). Supplementation of sows with L-carnitine during pregnancy and lactation improves growth of the piglets during the suckling period through increased milk production. J Nutr.

[22] Nissen S, Faidley TD, Zimmerman DR, Izard R, Fisher CT (1994). Colostral milk fat percentage and pig performance are enhanced by feeding the leucine metabolite beta-hydroxy-beta-methyl butyrate to sows. J Anim Sci.

[23] Yonamine C, Teixeira S, Campello R, Gerlinger‐Romero F, Rodrigues C, Guimarães‐Ferreira L (2014). Beta hydroxy beta methylbutyrate supplementation impairs peripheral insulin sensitivity in healthy sedentary Wistar rats. Acta Physiol.

[24] Davis TA, Fiorotto ML (2009). Regulation of muscle growth in neonates. Curr Opin Clin Nutr.

[25] Verstegen MW, Mesu J, van Kempen GJ, Geerse C (1985). Energy balances of lactating sows in relation to feeding level and stage of lactation. J Anim Sci.

[26] van den Brand H, Heetkamp MJ, Soede NM, Schrama JW, Kemp B (2000). Energy balance of lactating primiparous sows as affected by feeding level and dietary energy source. J Anim Sci.

[27] Kao M, Columbus DA, Suryawan A, Steinhoff-Wagner J, Hernandez-Garcia A, Nguyen HV, et al. Enteral *β*-hydroxy-*β*-methylbutyrate supplementation increases protein synthesis in skeletal muscle of neonatal pigs. Am J Physiol-Endoc M. 2016;310(11): E1072–84. 10.1152/ajpendo.00520.2015 PMC4935142 27143558

[28] Tatara MR, Krupski W, Tymczyna B, Studzinski T (2012). Effects of combined maternal administration with alpha-ketoglutarate (AKG) and beta-hydroxy-beta-methylbutyrate (HMB) on prenatal programming of skeletal properties in the offspring. Nutr Metab.

[29] Tatara MR, Sliwa E, Krupski W (2007). Prenatal programming of skeletal development in the offspring: effects of maternal treatment with beta-hydroxy-beta-methylbutyrate (HMB) on femur properties in pigs at slaughter age. Bone.

[30] Rehfeldt C, Tuchscherer A, Hartung M, Kuhn G (2008). A second look at the influence of birth weight on carcass and meat quality in pigs. Meat Sci.

[31] Wimmers K, Ngu N, Jennen D, Tesfaye D, Murani E, Schellander K (2008). Relationship between myosin heavy chain isoform expression and muscling in several diverse pig breeds. J Anim Sci.

[32] Men XM, Deng B, Xu ZW, Tao X, Qi KK (2013). Age-related changes and nutritional regulation of myosin heavy-chain composition in longissimus dorsi of commercial pigs. Animal.

[33] Rehfeldt C, Adamovic I, Kuhn G (2007). Effects of dietary daidzein supplementation of pregnant sows on carcass and meat quality and skeletal muscle cellularity of the progeny. Meat Sci.

[34] Wen W, Chen X, Chen D, Yu B, Luo J, Huang Z (2016). Cloning and functional characterization of porcine Sox6. Turk J Biol.

[35] Quiat D, Voelker KA, Pei J, Grishin NV, Grange RW, Bassel-Duby R (2011). Concerted regulation of myofiber-specific gene expression and muscle performance by the transcriptional repressor Sox6. Proc Natl Acad Sci U S A.

